# Comparative DCMS analysis reveals a novel breed-specific X-linked selection signature in Changthangi sheep

**DOI:** 10.3389/fgene.2026.1774902

**Published:** 2026-05-19

**Authors:** Sapna Nath, Satish Kumar Illa, Vinod Kumar Yata, Rishikesh Shukla, Narasaiah Kolliputi

**Affiliations:** 1 College of Veterinary Science, Livestock Farm Complex, Sri Venkateswara Veterinary University, Garividi, India; 2 Livestock Research Station, Sri Venkateswara Veterinary University, Garividi, India; 3 Department of Biotechnology, School of Allied Healthcare Sciences, Malla Reddy University, Hyderabad, Telangana, India; 4 Department of Biotechnology, GLA University, Mathura, India; 5 Division of Allergy and Immunology, Department of Internal Medicine, USF Morsani College of Medicine, Tampa, FL, United States

**Keywords:** de-correlated composite of multiple signals (DCMS), high altitude adaptation, Indian indigenous sheep, selection signatures, X chromosome

## Abstract

**Introduction:**

The X chromosome contributes to important adaptive and fitness-related traits in livestock, but it has received relatively limited attention in sheep genomics, particularly in studies of indigenous Indian breeds.

**Methods:**

We utilised the de-correlated composite of multiple signals (DCMS) approach, which integrates five individual statistics including FST, H1, H12, Tajima’s D, and π, to identify selection signatures within breeds on the X chromosome in Changthangi (n = 29), Deccani (n = 24), and Garole (n = 26) sheep, genotyped with the Illumina OvineSNP50 BeadChip. Candidate genes and quantitative trait loci (QTLs) in significant regions (FDR <0.05) were annotated using the GALLO package, followed by prioritisation and visualisation through protein–protein interaction networks.

**Results:**

Our analysis identified a significant genomic region on the X chromosome (∼55.9–57.2 Mb; ∼1.396 Mb) in Changthangi sheep, whereas no comparable signal was detected in Deccani or Garole. This region includes 31 genes, with the top-prioritized genes being *OTUD5*, *CACNA1F*, *ZNF182*, and *GRIPAP1*. The QTL annotation indicated enrichment for traits related to milk production, body weight, and reproduction, while protein-protein interaction networks identified *OTUD5* as a prioritized and relatively well-connected node within the candidate interval.

**Conclusion:**

This study identifies a breed-specific X-linked candidate selection region in Changthangi sheep and supports the view that X-linked variation may contribute to local adaptation at high altitudes. These findings highlight the value of within-breed composite approaches for investigating the X chromosome in indigenous sheep populations and provide a basis for future validation of candidate genes relevant to adaptation and breeding.

## 1 Introduction

Genomic variation in livestock populations is shaped by evolutionary forces such as natural selection, mutation, recombination, and genetic drift (Oleksyk et al., 2010; Qanbari and Simianer, 2014). Selection acting on advantageous alleles leaves recognizable genomic signatures, including reduced genetic diversity, increased haplotype homozygosity, and shifts in allele frequency spectra (Smith and Haigh, 1974; Vitti et al., 2013). Identifying these signatures provides important insights into the molecular basis of economically important and adaptive traits, including production, reproduction, disease resistance, survival, and environmental resilience. Such information can support sustainable breeding strategies in domesticated species.

The X chromosome is increasingly recognized as an important contributor to phenotypic variation in livestock because it harbors genes involved in reproduction, survival, immunity, and adaptation. However, compared with autosomes, the X chromosome has received relatively limited attention in livestock genomics, partly because of its distinct inheritance pattern and analytical complexity (Cunningham et al., 2022). This relative neglect may limit our understanding of the genetic architecture of important adaptive and reproductive traits and may reduce the accuracy of genomic evaluation in breeding programs (Su et al., 2014). Previous studies in livestock have also shown that X-linked loci can influence sex-limited reproductive and fertility traits (Fortes et al., 2020; Olasege et al., 2024), further supporting the biological importance of the X chromosome in livestock genomics. Recent studies in livestock, including sheep, have highlighted the contribution of X-linked regions to local adaptation and fitness-related traits (Shihabi et al., 2022; Rajawat et al., 2024; Goli et al., 2025).

Several statistical approaches have been developed to detect genomic signatures of selection. Within-population methods such as Tajima’s D, nucleotide diversity (π), and haplotype-based statistics capture different aspects of allele frequency distortion and haplotype structure, (Nei and Li, 1979; Tajima, 1989; Voight et al., 2006). whereas between-population methods such as fixation index (FST) and cross-population extended haplotype homozygosity (XP-EHH) are commonly used to identify differentiated regions among breeds (Weir and Cockerham, 1984; Holsinger and Weir, 2015; Gautier et al., 2017). Because individual statistics reflect only part of the selection process, composite approaches can improve robustness and resolution. The de-correlated composite of multiple signals (DCMS) framework integrates several univariate statistics into a single measure while accounting for the correlation among them, thereby increasing power to detect candidate selection regions.

India possesses rich sheep genetic resources adapted to diverse ecological zones, ranging from the cold arid Himalayan region to semi-arid plateau environments and saline coastal ecosystems (LivestockCensus, 2019). These contrasting production systems have contributed to marked breed-specific phenotypic and adaptive differences (Acharya, 1982; Bhatia and Arora, 2005).

Changthangi sheep, reared in the high-altitude cold desert of Ladakh, are adapted to hypoxia, extreme cold, and poor forage availability. In contrast, Deccani sheep are adapted to semi-arid conditions of the Deccan plateau, while Garole sheep are known for reproductive efficiency and adaptation to the saline ecosystem of the Sundarbans. These contrasting environments make these breeds an informative model for studying local adaptation. Recent work on Indian sheep has investigated X-chromosome selection signatures using cross-population approaches, particularly XP-EHH, and reported candidate X-linked regions associated with Changthangi adaptation (Goli et al., 2025). However, cross-population comparisons may overlook within-breed signatures that reflect breed-specific selective sweeps. In the present study, we applied the DCMS framework to investigate within-breed selection signatures on the X chromosome in Changthangi, Deccani, and Garole sheep. We hypothesized that this approach would reveal breed-specific X-linked signals not captured by cross-population methods. Therefore, the objectives of this study were to: (i) identify within-breed selection signatures on the X chromosome in the three indigenous Indian sheep breeds using DCMS; (ii) annotate genes and QTLs within significant candidate regions; and (iii) investigate functional relationships among candidate genes to better understand the X-linked architecture of local adaptation, with particular emphasis on high-altitude adaptation in Changthangi sheep.

## 2 Materials and methods

### 2.1 Animals and genotyping

This study investigated X-chromosome variation in three Indian sheep breeds: Changthangi (CHA, n = 29), Deccani (IDC, n = 24), and Garole (GAR, n = 26). Genotype data were generated using the Illumina OvineSNP50 BeadChip, a medium-density SNP array, and were obtained from the WIDDE database (Sempéré et al., 2015). Quality control was performed using PLINK v1.9. SNPs were filtered based on minor allele frequency (MAF <0.05), Hardy–Weinberg equilibrium (p < 0.0001), SNP missingness (>0.10), and individuals with excessive missing genotype data (>0.10). The physical positions of SNPs were mapped to the Rambouillet reference genome assembly ARS-UI_Ramb_v3.0. The X-chromosome SNP marker information used for downstream analysis is provided in Supplementary Table S3.

### 2.2 Principal component analysis (PCA)

Principal component analysis (PCA) was performed using the R package SNPRelate to summarise genomic variation and assess population structure among the three breeds (Zheng et al., 2012). PCA was conducted on the filtered SNP dataset, and the first two principal components were used to visualize breed-level genetic differentiation.

### 2.3 De-correlated composite of multiple selection signals

To identify candidate selection signals on the X chromosome, we applied the de-correlated composite of multiple signals (DCMS) approach, which integrates multiple univariate statistics while accounting for their correlation structure. Five univariate statistics were incorporated into the DCMS framework: fixation index (FST), haplotype homozygosity (H1), modified haplotype homozygosity (H12), Tajima’s D, and nucleotide diversity (π). Together, these statistics capture complementary features of population differentiation, haplotype structure, and allele-frequency spectra. Following Yurchenko et al. (2018), the DCMS statistic for locus l was calculated by combining the *p*-values of the individual statistics while accounting for their correlation.

$${DCMS}_{l\;}=\sum_{t=1}^{n}\frac{\log\lfloor \frac{1-P_{lt}}{P_{lt}}\rfloor }{\sum_{i=1}^{n}\left|r_{it}\right|}$$

where $p_{lt}$ is the *p*-value at locus l for statistic t , $r_{it}$ is the correlation between the i th and t th statistics, and n is the total number of statistics included in the DCMS framework. Thus, the denominator acts as a weight factor that reduces the contribution of highly correlated statistics and helps avoid inflation caused by non-independence among component tests (Lotterhos et al., 2017).

Genome-wide *p*-values for each component statistic were first obtained from their empirical distributions. Pairwise correlations among the genome-wide smoothed vectors of FST, H1, H12, Tajima’s D, and π were then estimated across all analysed X-chromosome loci and assembled into a correlation matrix. This matrix was used as input for the DCMS calculation so that highly correlated statistics contributed proportionally less to the final composite score. The resulting DCMS values were then fitted to a normal distribution, and *p*-values were obtained using the pnorm function. Multiple testing was controlled by converting these *p*-values to *q-*values using the Benjamini–Hochberg false discovery rate procedure implemented in R using the *p*.adjust function.

The DCMS workflow was implemented in R using the MINOTAUR (Verity et al., 2017), rrcovNA (Todorov et al., 2011), and MASS (Venables and Ripley, 2002) packages, and multiple testing was controlled using the p.adjust function in R (R Core Team, 2020). A schematic overview of the DCMS workflow, including intermediate analytical steps and downstream interpretation, is provided in Supplementary Figure S1. The breed-wise correlation matrices of the five DCMS component statistics used in the integration step are provided in Supplementary Tables S8a–S8c.

### 2.4 Computation of univariate statistics

The five component statistics used in the DCMS framework were computed as follows. *F*ST values were calculated in PLINK v1.9 using the--fst and--within options (Chang et al., 2015). Negative *F*ST values are set to zero, and the resulting values are smoothed using the runmed function in R (R Core Team, 2020). The Tajima’s *D* was calculated using VCFtools with a 300-kb window size (Danecek et al., 2011). Nucleotide diversity (π) was also estimated using VCFtools, and π values were subsequently smoothed using the runmed function in R (Danecek et al., 2011; R Core Team, 2020).

Haplotype-based statistics H1 and H12 were calculated using the H12_H1H2.py script (Garud et al., 2015). Prior to haplotype-based analysis, genotypes were phased using SHAPEIT (Delaneau et al., 2012), and phased haplotypes were converted into the format required for the H12/H1H2.py program. The statistics were calculated using the program’s default parameters.

Together, these statistics provided complementary information on population differentiation, haplotype homozygosity, and local sequence diversity, which were subsequently integrated in the DCMS framework. Prior to DCMS integration, the component statistics were smoothed where appropriate, and the smoothed genome-wide vectors were used for correlation estimation and downstream DCMS computation.

### 2.5 Identification of candidate regions, genes, and QTLs

Candidate selection regions were identified from the DCMS results using a false discovery rate approach. SNPs with q-values below 0.05 were considered significant, and contiguous significant SNPs were grouped into candidate regions. Genes and quantitative trait loci (QTLs) located within significant regions were annotated using the GALLO package (v4.2.0) (Fonseca et al., 2020). Gene annotation was performed using the file Ovis_aries.ARS-UI_Ramb_v3.0.115.gtf.gz, corresponding to the Rambouillet reference genome assembly ARS-UI_Ramb_v3.0, with annotation data obtained from Ensembl release 115 (Cunningham et al., 2022). QTL coordinates were obtained from the Sheep QTL database (Hu et al., 2016). To avoid coordinate inconsistency, all annotations were harmonized to ARS-UI_Ramb_v3.0, which was also used for SNP mapping.

### 2.6 Gene prioritization and network analysis

Genes within the significant X-chromosome region were further evaluated using an exploratory prioritisation framework intended to highlight biologically relevant candidates for downstream interpretation. Annotation data, including gene name, Ensembl ID, gene biotype, chromosome position, and genomic coordinates, were retrieved from Ensembl BioMart using the biomaRt package for the *Ovis aries* ARS-UI_Ramb_v3.0 genome release 115 (Cunningham et al., 2022; Durinck et al., 2009).

Candidate genes were ranked using three criteria:

- gene biotype, with preference given to protein-coding genes;
- overlap with annotated QTLs; and
- relative gene span within the candidate region.

These criteria were used as a heuristic aid for candidate selection rather than as a formal inferential model. Because gene length may bias ranking toward longer genes, prioritization results were interpreted together with functional annotation, network connectivity, and literature evidence, rather than in isolation.

To explore functional relationships among annotated genes, a protein-protein interaction (PPI) network was generated and visualized in R using the igraph and ggraph packages. Genes prioritized by the exploratory ranking procedure were highlighted within the network to facilitate biological interpretation of the candidate region (Harrow et al., 2012; Moreau and Tranchevent, 2012; Lawrence et al., 2013; R Core Team, 2020).

## 3 Results

### 3.1 Quality control of genotypes

After quality control, 10 SNPs were removed because of missing genotype data, 39 SNPs failed the Hardy-Weinberg equilibrium test, and 139 SNPs were excluded based on minor allele frequency filtering. In addition, five individuals were removed because of mixed samples or excessive missingness. A final dataset comprising 1,044 X-chromosome SNPs from 74 animals was retained for downstream analysis. Detailed sample- and SNP-level quality control summaries are provided in Supplementary Table S1, and chromosome-wise SNP counts before and after quality control are provided in Supplementary Table S2.

### 3.2 Principal component analysis (PCA)

**FIGURE 1:**
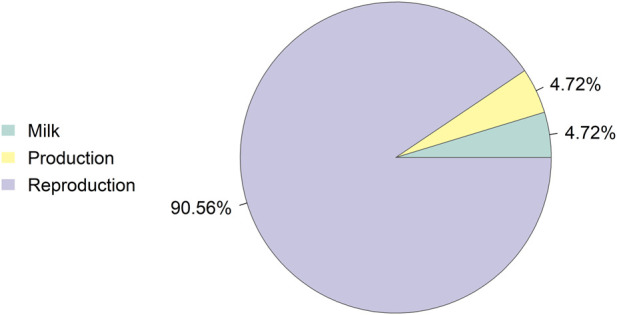
Principal component analysis (PCA) of X-chromosome SNP variation in Changthangi (CHA), Garole (GAR), and Deccani (IDC) sheep. The first principal component (PC1) explained 15.74% of the variation, and the second principal component (PC2) explained 10.9%, showing clear breed-wise clustering.

Principal component analysis of the filtered X-chromosome SNP dataset showed clear breed-wise clustering of Changthangi, Deccani, and Garole sheep, indicating marked genetic differentiation among the three populations. The first principal component explained 15.74% of the total variation, while the second principal component explained for 10.9%. Changthangi sheep formed a distinct cluster separate from Deccani and Garole, consistent with its unique evolutionary history and adaptation to the high-altitude environment. These results are shown in Figure 1.

### 3.3 De-correlated composite of multiple selection signals (DCMS)

**FIGURE 2:**
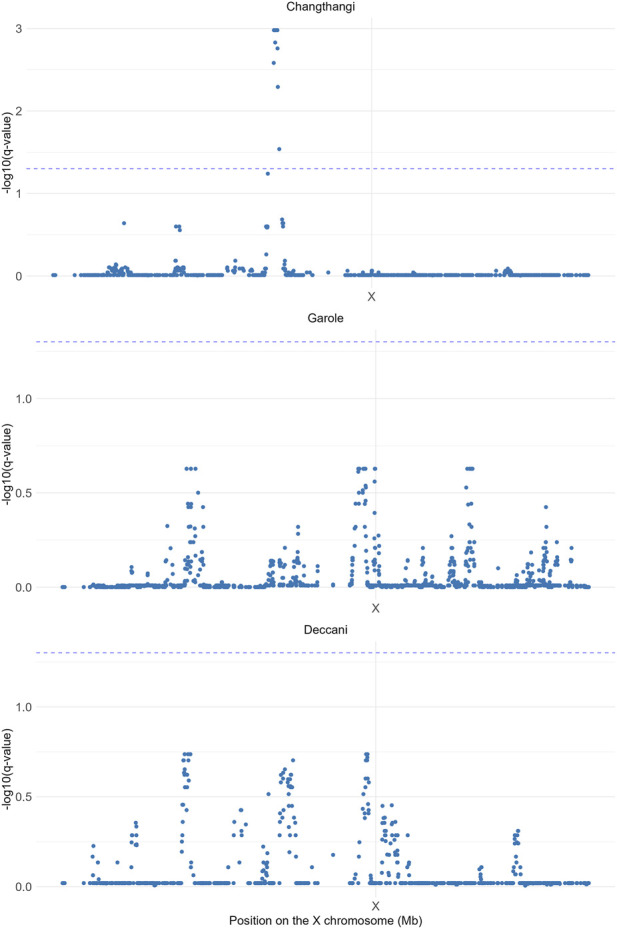
DCMS-based Manhattan plot of X-chromosome selection signatures in Changthangi (CHA), Garole (GAR), and Deccani (IDC) sheep. The x-axis represents genomic position on the X chromosome (Mb), and the y-axis represents–log10 (q-value). Significant signals were detected only in Changthangi sheep within the 55.79–57.18 Mb interval.

The DCMS analysis identified a significant candidate selection region in Changthangi sheep, extending from 55.79 to 57.18 Mb on the X chromosome, spanning approximately 1.396 Mb. No comparable signal was observed in Deccani or Garole sheep. The corresponding Manhattan plot is presented in Figure 2. The DCMS-identified genomic regions are summarized in Table 1.

**TABLE 1:** Genomic regions on the X chromosome under selection identified by DCMS.

S.No.	Breed	Total size (kb)	Total no. of genes	*P*-value	*Q*-value
01	Chanthangi (CHA)	1395.78	31	6.35e-06	0.0010
02	Garole (GAR)	-	-	-	-
03	Deccani (IDC)	-	-	-	-

### 3.4 Gene and QTL annotation of the candidate region

**FIGURE 3:**
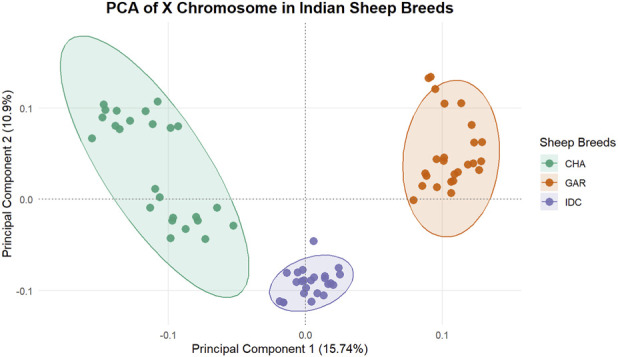
QTL classes annotated on the x chromosome of Changthangi sheep.

Annotation of the significant Changthangi region identified 31 genes located within the 55.79-57.18 Mb interval on the X chromosome. Among these, *OTUD5* was prioritised as the top candidate gene in the region followed by *CACNA1F, ZNF182, GRIPAP1,* and *GPKOW*. In addition, the candidate interval overlapped QTLs associated with traits including offspring number, milk yield, and body weight. The annotated genes and corresponding genomic information are summarized in Table 2. Detailed significant SNP outputs, candidate-region summaries, gene annotation results, and gene-prioritization outputs are provided in Supplementary Tables S4–S7, while the distribution of QTL classes identified in the candidate region is shown in Figure 3. The QTL annotations for milk, production, and reproduction traits in the candidate X-chromosome region are presented in Table 3.

**TABLE 2:** Comprehensive list of annotated genes with genomic start and end positions, and gene length.

Rank	Gene name	Ensembl gene ID	Start position	End position	Gene length (bp)
1	*OTUD5*	ENSOARG00020089132	56128072	56155571	27500
2	*CACNA1F*	ENSOARG00020091235	55917101	55944263	27163
3	*ZNF182*	ENSOARG00020086223	56726068	56750267	24200
4	*GRIPAP1*	ENSOARG00020083765	56093137	56114263	21127
5	*GPKOW*	ENSOARG00020092905	55990252	56011136	20885
6	*HDAC6*	ENSOARG00020096440	56240983	56259595	18613
7	*ELK1*	ENSOARG00020043129	57177991	57193900	15910
8	*CCDC22*	ENSOARG00020085369	55900145	55915777	15633
9	*WDR13*	ENSOARG00020079723	56459312	56473394	14083
10	*TBC1D25*	ENSOARG00020064124	56493754	56507304	13551
11	*SUV39H1*	ENSOARG00020076175	56387585	56400257	12673
12	*PORCN*	ENSOARG00020081083	56514614	56526040	11427
13	*TFE3*	ENSOARG00020079611	56071375	56082362	10988
14	*GLOD5*	ENSOARG00020062045	56283760	56293390	9631
15	*CCDC120*	ENSOARG00020083734	56039566	56048814	9249
16	*SLC38A5*	ENSOARG00020081678	56575541	56584723	9183
17	*PRICKLE3*	ENSOARG00020083037	55962258	55971249	8992
18	*SLC35A2*	ENSOARG00020048251	56165472	56173036	7565
19	*WAS*	ENSOARG00020091399	56404313	56411820	7508
20	*FOXP3*	ENSOARG00020097836	55893503	55900971	7469
21	*UXT*	ENSOARG00020047085	57169979	57176936	6958
22	*GATA1*	ENSOARG00020072751	56267707	56274495	6789
23	*PQBP1*	ENSOARG00020092831	56172166	56178629	6464
24	*FTSJ1*	ENSOARG00020073386	56562803	56569238	6436
25	*PIM2*	ENSOARG00020081088	56158261	56163723	5463
26	*WDR45*	ENSOARG00020075562	56029975	56035257	5283
27	*MAGIX*	ENSOARG00020077247	55976619	55981487	4869
28	*PCSK1N*	ENSOARG00020078901	56230716	56235346	4631
29	*TIMM17B*	ENSOARG00020073521	56178371	56182653	4283
30	*ERAS*	ENSOARG00020058301	56236277	56239732	3456
31	*RBM3*	ENSOARG00020076500	56479962	56482943	2982

**TABLE 3:** Quantitative trait loci (QTL) annotations in the X chromosome region (∼55.9–57.2 Mb) of Changthangi sheep.

Chromosome	Trait class	Start position	End position	Trait	Marker ID	Number of QTLs
X	Milk	50223527	50223531	180-day milk yield	rs410680220	11
X	Production	63727222	63727226	Body weight	rs425809376	11
X	Reproduction	46145421	65172048	Offspring number	rs429304767, rs399006822, rs415551863, rs426009843, rs588705860, rs596745912, rs418740875, rs412881200, rs429350392, rs413777698, rs415759946, rs425194841, rs399855449, rs423387214, rs421879546, rs422072495, rs417544944, rs426139622, rs405487023, rs402306037	211

### 3.5 Protein-protein interaction network analysis

**FIGURE 4:**
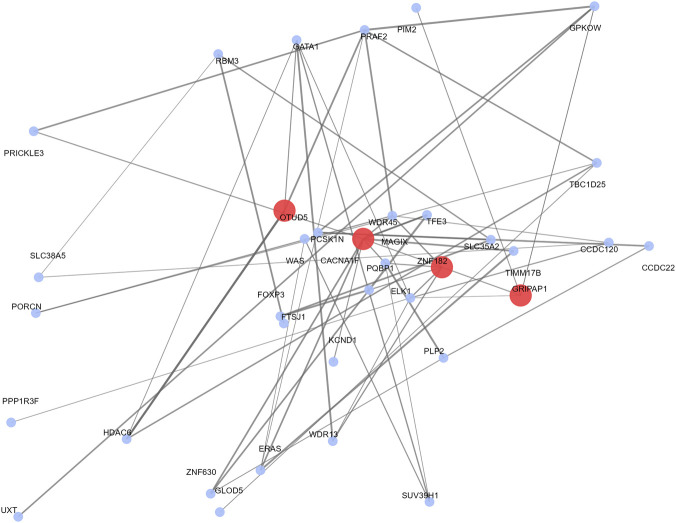
PPI network highlighting prioritized x chromosome genes in Changthangi.

Protein–protein interaction analysis of genes in the selected X-chromosome interval identified *OTUD5* as a relatively well-connected node in the network. The network supported functional relatedness among several genes within the candidate interval, including *CACNA1F* and *HDAC6*, but did not provide evidence for a direct interaction between *OTUD5* and *FOXP3*. The interaction network is presented in Figure 4.

## 4 Discussion

This study applied the de-correlated composite of multiple signals (DCMS) framework to investigate within-breed selection signatures on the X chromosome in three indigenous Indian sheep breeds. A single prominent candidate region was identified in Changthangi sheep at approximately 55.79–57.18 Mb, whereas no comparable signal was detected in Deccani or Garole. This result indicates that the X chromosome harbors a breed-specific signal of selection in Changthangi and supports the view that X-linked variation may contribute to local adaptation in sheep populations exposed to extreme environments. In the present case, the most plausible selective context is the high-altitude, cold, and nutritionally constrained environment in which Changthangi sheep have evolved.

The PCA results further support genetic differentiation among the three breeds based on X-chromosome variation. Changthangi formed a distinct cluster relative to Deccani and Garole, consistent with its ecological and historical separation. This pattern is compatible with the interpretation that X-linked loci in Changthangi have been shaped by breed-specific selective pressures. However, PCA alone does not identify causal loci; rather, it provides complementary evidence that the three breeds are genetically differentiated on the X chromosome.

A central finding of this study is the candidate interval containing *OTUD5* and a connected set of genes including *CACNA1F, HDAC6*, and *FOXP3.* Among these, *OTUD5* emerged as the most strongly prioritised and one of the more connected genes in the protein–protein interaction network, making it a plausible functional hub within the selected region rather than simply one gene among many annotated candidates. *OTUD5* encodes an *OTU*-family deubiquitinase and has been implicated in several core regulatory processes, including innate immune signalling, DNA-damage responses, and chromatin-associated transcriptional control (Guo et al., 2021; de Vivo et al., 2019; Beck et al., 2021). In particular, *OTUD5* has been shown to stabilize STING and promote antiviral innate immune signalling, while also contributing to genome-stability pathways and transcriptional regulation at damaged chromatin (Guo et al., 2021; de Vivo et al., 2019). These functions support the interpretation that *OTUD5* may act as a regulatory integrator in cellular stress-response pathways. In Changthangi sheep, such functions are consistent with a plausible role in physiological resilience under chronic environmental stress, including hypoxia and cold exposure, although this interpretation remains inferential and requires direct functional validation in ovine systems.

The associated genes in this interval support a network-based interpretation of the Changthangi-specific signal. *CACNA1F* is involved in calcium-dependent signalling and may contribute to neuroendocrine and cellular stress responses, and has also been implicated in reproductive seasonality and environmental responsiveness in sheep (McRory et al., 2004; Du et al., 2022). HDAC6 is linked to cytoskeletal regulation and stress-related cellular remodelling, with known roles in the deacetylation of α-tubulin and actin-associated proteins that may influence cellular adaptation under stress (Boyault et al., 2007). *FOXP3* remains a biologically relevant positional candidate because of its role in immune tolerance and reproductive immunology (Buckner and Ziegler, 2008; Ibrahim et al., 2023), although the PPI network shown in Figure 4 does not support a direct interaction between *FOXP3* and *OTUD5*. Together, these genes suggest that the selected interval may influence interconnected processes related to immune balance, signal transduction, and physiological resilience under chronic environmental stress, including hypoxia and cold exposure.

Although the selected interval overlapped QTLs associated with offspring number, milk yield, and body weight, these findings do not necessarily contradict an adaptation-based interpretation. As shown in Figure 3, the annotated QTL classes within the Changthangi candidate region were mainly related to reproduction, milk production, and body weight, supporting the view that this X-chromosomal interval may affect multiple fitness-related traits under environmental stress. In high-altitude systems, trade-offs involving energy allocation, reproduction, growth, and survival are biologically plausible. Therefore, the presence of economic-trait QTLs within the selected region may reflect pleiotropic or fitness-related effects under environmental stress rather than traits unrelated to adaptation. Similar links between X-linked loci and prolificacy, milk-related traits, and growth have been reported in sheep and other livestock studies (Chantepie et al., 2020; Li et al., 2020; Raziq et al., 2024).

Our results differ from those reported by Goli et al. (2025), who used a cross-population XP-EHH framework to investigate X-linked selection signatures in Indian sheep. This difference is not necessarily contradictory. XP-EHH and DCMS capture different aspects of selection and operate under different analytical assumptions. XP-EHH is designed to detect extended haplotype differences between populations, whereas DCMS integrates multiple within-population statistics and may be more sensitive to breed-specific selective sweeps that are not strongly expressed as cross-population contrasts. The present findings therefore complement, rather than invalidate, previous work and highlight the value of combining within-breed and cross-population approaches when studying adaptation in indigenous livestock populations.

This study also has important limitations. First, the analysis was based on medium-density SNP array data, and the X chromosome contained a relatively limited number of polymorphic markers after quality control. Second, the candidate-gene prioritization strategy is exploratory and should not be interpreted as definitive evidence of biological importance. Third, functional inference for several genes relied on orthologous evidence from other mammals because direct ovine functional evidence remains limited. Finally, the conclusions are based on statistical signatures of selection and annotation overlap and therefore require validation through denser genotyping, sequencing, transcriptomic analysis, or experimental functional studies. Future studies should validate these findings using higher-resolution whole-genome sequence data, including publicly available international sheep resources such as the ISGC “3000 Sheep Genomes” project.

Overall, the present study identifies a Changthangi-specific X-chromosome candidate region and supports the hypothesis that X-linked loci contribute to high-altitude adaptation in indigenous Indian sheep. The combination of a breed-specific DCMS signal, functional annotation, QTL overlap, and network analysis points to an interval containing OTUD5 and associated genes as a promising target for future investigation. These findings add to the growing evidence that the X chromosome should not be neglected in studies of ovine adaptation and may provide useful biological targets for future breeding and conservation strategies.

## 5 Conclusion

In summary, this study applied the de-correlated composite of multiple signals (DCMS) framework to investigate within-breed selection signatures on the X chromosome in three indigenous Indian sheep breeds. The analysis identified a significant selection signature located at 55.9–57.2 Mb, which is exclusively present in the high-altitude Changthangi breed, while absent in the Deccani and Garole breeds. This candidate region contains 31 annotated genes, among which OTUD5 and a small set of prioritized genes emerged as biologically plausible candidates for further investigation. QTL annotation further indicated overlap with reproduction, milk yield, and body-weight traits. No comparable signal was detected in Deccani or Garole, supporting the breed-specific nature of the Changthangi X-chromosome signal. These findings support the view that X-linked variation may contribute to local adaptation in sheep, particularly under the hypoxic, cold, and nutritionally constrained conditions experienced by Changthangi sheep. More broadly, this study highlights the value of investigating the X chromosome, which remains relatively understudied in livestock genomics, and demonstrates the usefulness of within-breed composite approaches for detecting adaptive signatures that may not be captured by cross-population methods. However, these findings are based on statistical evidence and annotation-based inference and therefore require validation through denser genotyping, sequencing, transcriptomic approaches, and functional studies in larger populations.

## Data Availability

Publicly available datasets were analyzed in this study. This data can be found here: http://widde.toulouse.inra.fr/widde/.
