# Supplementary material for: Comparative DCMS analysis reveals a novel breed-specific X-linked selection signature in Changthangi sheep
Source: Front Genet. 2026 May 19;17:1774902. doi: 10.3389/fgene.2026.1774902 (PMC13225777; doi:10.3389/fgene.2026.1774902)
Supplement: Supplementary file 2 [file DataSheet1.docx]

***Supplementary Material***


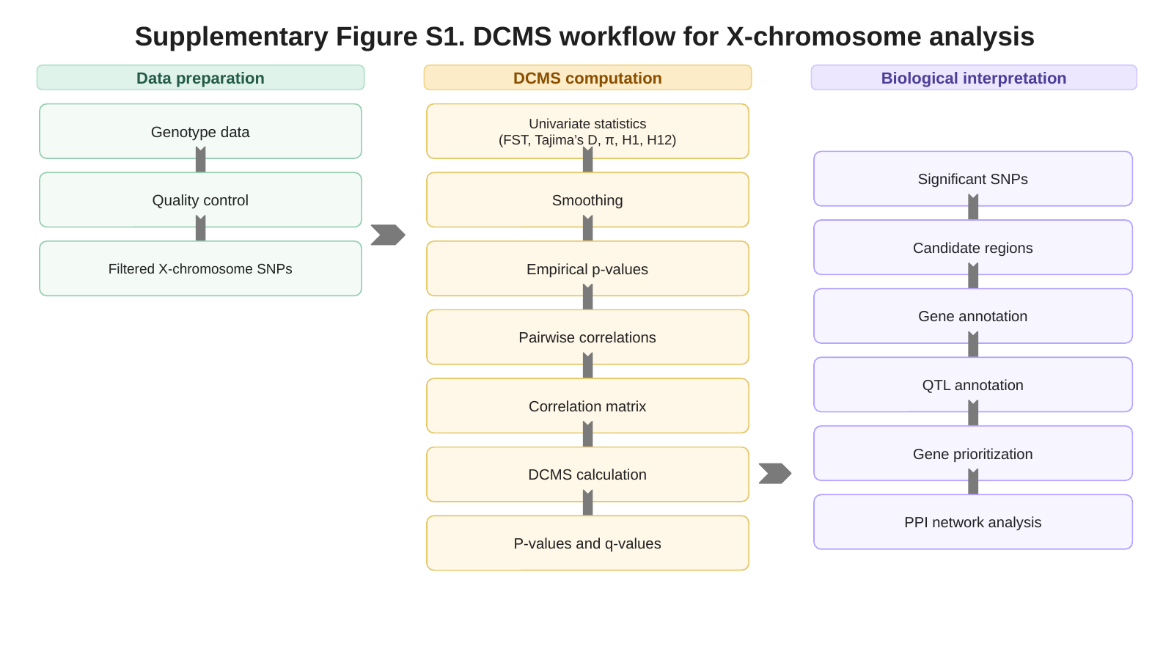


**Supplementary Figure S1. Workflow of the decorrelated composite of multiple signals (DCMS) analysis for detection of genomic regions under selection.** The schematic illustrates the stepwise analytical framework employed in the study. Initially, multiple selection statistics—including fixation index (FST), haplotype-based statistics (H1 and H12), Tajima’s D, and nucleotide diversity (π)—were computed across the genome. These statistics were subsequently standardized and integrated using the DCMS approach, which accounts for the covariance structure among individual test statistics to generate a composite score. The resulting DCMS values were used to identify candidate genomic regions under selection. Downstream analyses included identification of significant loci, annotation of candidate genes, and functional interpretation.
